# Optimisation of a Mouse Model of Cerebral Ischemia-Reperfusion to Address Issues of Survival and Model Reproducibility and Consistency

**DOI:** 10.1155/2022/7594969

**Published:** 2022-07-06

**Authors:** Zhenqian Liu, Mo Chen, Xu Duan, Yujia Zhai, Bin Ma, Zuowei Duan, Jiang Xu, Haiyan Liu

**Affiliations:** Department of Neurology, The Second Affiliated Hospital of Xuzhou Medical University, Xuzhou 221000, China

## Abstract

Middle cerebral artery occlusion (MCAO) induced brain ischemia-reperfusion model in Mice is essential for understanding the pathology of stroke and investigating potential treatments, in which a variety of methods may be employed to block the middle cerebral artery (MCA), the most common being through the insertion of a monofilament; however, in vivo ischemia-reperfusion models are associated, particularly in mice, with high variability in lesion volume and high mortality. We aimed to optimise a mouse model of cerebral ischemia-reperfusion, addressing issues of mouse survival, model reproducibility, and consistency. The model was optimised in two ways: first, insert the monofilament directly through the internal carotid artery rather than through the external or common carotid artery, and second, by extending the length of the silicone coating on the monofilament, the length of the silicone coating enables embolization of the beginning of the middle cerebral artery, as well as the anterior cerebral artery and part of the posterior communicating artery. *Results: *We assessed various parameters, including blood flow changes in the middle cerebral artery, stability of the infarct area, correlation between infarct volume percentages and neurological deficit scores, mortality, weight changes, and wellbeing. We found that optimisation of the surgical procedure may improve mouse wellbeing and reduce mortality, through reduced weight loss and decrease the variability. In conclusion, we suggest that the optimisation of the model is superior for the study of both short and long-term outcomes of ischemic stroke. These results have considerable implications on stroke model selection for researchers.

## 1. Introduction

Stroke is one of the leading causes of adult mortality and disability [1]. Ischemic strokes in humans arise most commonly from blockage of the middle cerebral artery (MCA) and its branches [2]. Stroke animal models play an essential role in the investigation of mechanisms and drug development [3]. Over the last four decades, a variety of animal stroke models, including the intraluminal MCA occlusion (MCAO) model [4, 5], photothrombosis model [6], craniotomy model [7], atherothrombotic model [8, 9], and embolic stroke model [10], have been developed. Intraluminal MCAO in mice is the most frequently used model of stroke as it allows the restoration of blood flow after the induction of focal ischemia thus mimicking human ischemic stroke, exhibiting a penumbra, being highly reproducible and having no craniotomy [11, 12]. Many efforts have been made to improve this model [4, 13–15], but the survival rate, success rate, and stability of the model remained unsatisfying and diverse among different individual studies [16]. Which may be due to the variation of lateral circulation and excessive vascular damage and the difficulty of the operation procedure [17, 18].

To allow for permit reperfusion the vessel through which the monofilament is inserted, which is either the common carotid artery (CCA) [19] or the external carotid artery (ECA) [20], remains permanently ligated. Permanent ligation of the CCA prevents restoration of blood flow through the internal carotid artery this structure has a high anatomical variability and hemodynamic abnormality [21]. The alternative is to insert the monofilament via the ECA, also known as the classical procedure [22], ECA transection in rats can induce ischemic lesions of the mastication and swallowing muscles [23, 24], if vascular supply to the ECA is compromised this has been shown, in rats, affecting drinking behaviours and increasing body weight loss post-MCAO, to have a detrimental effect on the animal's wellbeing [25]. New research suggests that post-MCAO mortality in mice is not primarily caused by ischemic brain damage, but secondarily by inadequate food and/or water intake [26].

The posterior communicating artery (PCoA) and the proximal segment of the anterior cerebral artery(ACA) collateral blood flow during occlusion may cause inconsistent infarction: variable residual flow within the MCA territory, which has been well characterized, [27, 28]. Thus, we investigated an optimised surgical approach in mice avoiding ECA, pterygo palatine artery (PPA) ligation, survival rate, and wellbeing can be improved in mice, extending the length of silicone coating on monofilament reduces the collateral blood supply from ACA and PCoA to MCA, variability within this model will be reduced.

## 2. Materials and Methods

### 2.1. Animals

The experimental procedures were approved by the Laboratory Animal Ethics Committee of Xuzhou Medical University (No. 201702w012). All procedures were carried out in accordance with the National Institutes of Health Guide for the Care and Use of Laboratory Animals (NIH Publications No. 8023, revised 2011).

**Figure 1:**
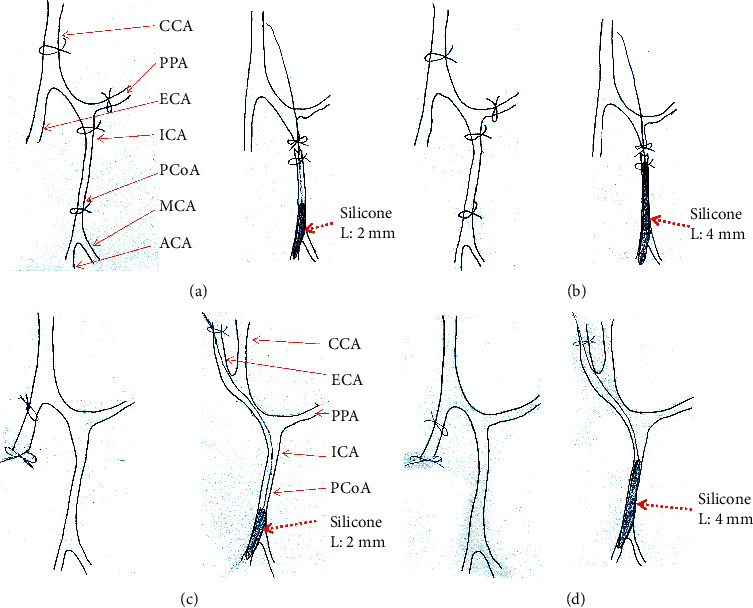
Schematic diagrams of the 4 MCAO procedures with large vessels and operating location: (a) ICA1 procedure, (b) ICA2 procedure, (c) ECA1 procedure, and (d) ECA2 procedure. Abbreviations: CCA, Common carotid artery; ECA, External carotid artery; ICA, Internal carotid artery; OA, Occipital artery; PPA, Pterygopalatine artery; PCoA, Posterior communicating artery.

A total of 120 Kunming (KM) male mice (Xuzhou Medical University Laboratory Animal Centre, China) at 10 weeks, weighing 20–23 g were used in our study. The mice were randomly divided into one of the following experimental groups: sham-operated group, internal carotid artery access group (ICA1 group) (Figure 1(a)), internal carotid artery access group + long silicone coated monofilament (ICA2 group) (Figure 1(b)), external carotid artery access group (ECA1 group) (Figure 1(c)), external carotid artery access group + long silicone coated monofilament (ECA2 group) (Figure 1(d)).

The animals were housed in a controlled environment with an ambient temperature of 25°C and relative humidity of 65%, under a 12 h light/dark cycle, and were allowed free access to water and food. Observations were conducted once per day before surgery and every 6 hours after surgery to monitor the health of the animals.

### 2.2. Surgery

#### 2.2.1. Anesthetic

Animals were anaesthetized with an intraperitoneal injection of ketamine and diazepam. A heating pad was employed to maintain the core temperature of 37 ± 0.5°C, measured with a rectal temperature probe. Before starting surgery, the depth of anesthesia was assessed by toe pinch. If no response was elicited, the surgery would be started.

#### 2.2.2. Measurement of Cerebral Blood Flow (CBF)

CBF in the MCA was monitored by using a laser Doppler flowmeter (LDF; PeriFlux system 5000; Perimed, Stockholm, Sweden), territory an incision (1 cm) was made on the skin overlying the calvarium, and the skin was pulled laterally to affix a microtip. The microtip was placed perpendicular to the surface of the right parietal skull (1 mm posterior and 5 mm lateral to the bregma). [29] only mice with 75% flow reduction after monofilament insertion were included in this study, We recorded MCA blood flow primarily preoperatively, at 0 minutes and 80 minutes after insertion of the monofilament.

#### 2.2.3. MCAO

The ICA1 and ICA2 groups were operated with a monofilament inserted from the ICA, the ECA1 and ECA2 groups were operated with a monofilament inserted from the ECA (Figure 1). The difference between groups 1 and 2 is the different lengths of the silicone coating on the monofilament, 2 mm for 1 and 4 mm for 2. The ICA2 performs an optimised procedure.

The mouse was placed on the operating table in a supine position under a stereo dissecting microscope, disinfection of fur with 75% ethanol.

*(1) The Classic MCAO Model*. (i) Vessel separation. after dissection of the carotid triangle, the common carotid artery (CCA), ECA, ICA, and occipital artery (OA) were identified. Cut into 20 mm segments with 6-0 sutures. place a permanent suture around the ECA, as far distally as possible, and another temporary suture on the ECA distal to the bifurcation (ii) Monofilament insertion. A temporary knot is tied in the proximal end of the CCA with a suture to prevent blood leakage, the ICA is clamped closed with a microarterial clip, and a small hole is cut between the permanent and temporary suture on the ECA. A 12 mm long 6-0 silicone coating monofilament (monofilament size 6-0, coating diameter 0.2 ± 0.01 mm, coating length 2 mm or 4 mm) is introduced into the ECA. The ECA distal to the permanent suture is completely severed, the free segment of the ECA is pulled outward and upward so that it runs parallel to the course of the ICA, and the monofilament is slowly fed downstream into the ICA. When the monofilament enters at a depth of 9-10 mm while resistance is felt, the suture on the ECA is tied tightly to secure the nylon monofilament and open the temporarily blocked CCA. (iii) Ligature suture. Remove the monofilament after 80 min and close the surgical incision layer by layer. (iv) Surgical care. All mice received subcutaneous buprenorphine (0.05 mg/kg; Temgesic, Schering-Plough Europe, Belgium) for pain relief at 1 h before surgery. The animals were maintained at 37 ± 0.5°C during and after surgery until they were fully recovered from anesthesia, when they were returned to their solitary cages in a heated (25–26°C) environment with free access to food and water.

**Figure 2:**
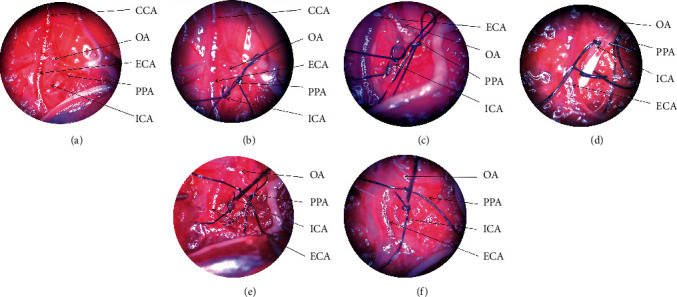
The surgical procedures of Optimised MCAO model. Notes: (1) Expose vital vessels by carefully separating the carotid triangle. (2) The thick OA emanating from the beginning of the ECA often forms a crossover with the ICA, eliminating the interference of the OA and accurately locating the PPA and ICA.

*(2) Optimised MCAO Model*. (i) Vessel separation. After dissection of the carotid triangle, CCA, ECA, ICA, OA, and PPA were identified (Figure 2(a)). (ii) Monofilament insertion. Temporary knots were tied in CCA, PPA with sutures place a permanent suture around the ICA, as far distally as possible, and another temporary suture on the ICA distal to the bifurcation (Figures 2(b) and 2(c)), a small hole is cut between the permanent and temporary suture on the ICA. A 12 mm long 6-0 silicone coating monofilament (monofilament size 6-0, coating diameter 0.2 ± 0.01 mm, coating length 2 mm or 4 mm) is introduced into the ICA (Figure 2(d)), and the monofilament is slowly fed downstream into the ICA. When the monofilament enters at a depth of 9-10 mm while resistance is felt, the suture on the ICA is tied tightly to secure the nylon monofilament (Figure 2(e)), The ICA distal to the permanent suture is completely severed and open the temporarily blocked CCA, PPA (Figure 2(f)), (iii) Ligature suture. Remove the monofilament and suture ICA permanent after 80 min, close the surgical incision layer by layer. (iv) Surgical care, Same as (A).

### 2.3. Neurological Severity Score (NSS)

NSS scores were performed on each group of mice at 24 h, 72 h, and 1 w postoperatively, respectively.

The tests include the evaluation of motor (raising the mouse by the tail, placing the mouse on the floor), sensory (placing test and proprioceptive test), reflex (reflex absence and abnormal movements), and balance (beam balance tests) deficits on a scale of 0 to 18 (0: normal score; 18: maximal deficit score) [30].

### 2.4. Measurement of Infarct Volume Percentage

24 h postoperative, mice were euthanized and brains collected for measurement of infarct size using 2,3,5-triphenyl tetrazolium chloride (TTC; Sigma, St. Louis, MO, USA) histology and digital image analysis, as previously described [31]. Their brains were subsequently removed and frozen at −80°C for 2 min, then cut into slices with a thickness of 2 mm from the cephalic to the caudal portion. Brain sections were incubated in 2% 2,3,5-TTC at 37°C for 15 min before imaging. The infarct volume percentage was corrected using standard methods (whole contralateral hemispheric volume-ipsilateral nonischemic hemispheric volume) and it was expressed as a percentage of the whole brain volume. Brain infarct volume percentages were measured and calculated using Image-Pro Plus 6.0 software.

### 2.5. Model Success Rate

The success rate of the model was calculated using an NSS score of 4 or more and the appearance of white infarct lesions on TTC staining as criteria for evaluating the success of the model, respectively, 24 h after surgery.

TTC solution is a clear, redox indicator that turns red only when applied to live cells. Brain tissue in the infarcted area appears white and any form of blood on the surface of the brain is considered a brain haemorrhage. We use the presence of white infarcted lesions as a criterion for successful modelling, and any locally dark red brain tissue is considered a model failure, as is the presence of brain haemorrhage.

### 2.6. Survival Rate

Calculated as the percentage of animals alive in each group 24 h, 72 h, 1 w postoperative.

### 2.7. Wellbeing Assessment

24 h, 72 h, and 1 w postoperatively, a general neurological scale (Clark general) was used to evaluate the wellbeing of mice [32]. The Clark general test addresses the hair, ears, eyes, posture, spontaneous activity, and epileptic behaviour of the animals. For each of the six general deficits measured, the scores in the six areas are summed to provide a total general score ranging from 0 to 28.

### 2.8. Weight Change

Mice were weighed before, 24 hours, 72 hours, and 1 w after the operation and compared to their preoperative body weight. Rate of weight change = (preoperative weight at each time point − postoperative weight)/preoperative weight.

### 2.9. Statistical Analysis

Parametric data are presented as the means ± SEM, and nonparametric data are presented as the medians ± interquartile ranges. Statistical analysis was performed using SPSS software (SPSS Inc., Chicago, IL, USA). Parametric data were analysed via one-way ANOVA, *t*-test and nonparametric data via Kruskal-Wallis ANOVA on ranks, followed by Dunn's posthoc testing. Correlations were analysed using Spearman's correlation. Model Success Rate and survival rate between the groups were compared using the Fisher exact test. The level of statistical significance was defined as *P* < 0.05.

## 3. Results

### 3.1. Cerebral Blood Flow Reduction (CBF)

**Figure 3:**
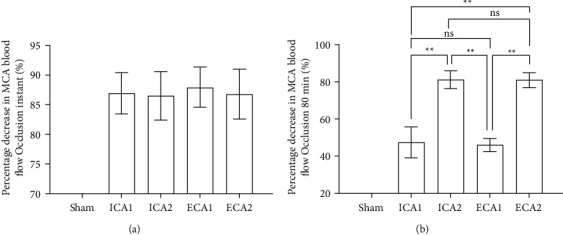
Percentage decrease in MCA blood flow. (a) No significant difference in the percentage decrease in MCA flow between the groups ICA1, ICA2, ECA1, and ECA2 after monofilament insertion, *P* > 0.05, *n* = 15. (b) 80 minutes after insertion of the monofilament, the percentage decrease in MCA flow was significantly greater in ICA1 and ECA1 than in ICA2 and ECA2. ^*∗*^*P* *<* 0.05, *n* = 15, while there was no significant difference between groups 1 and 2 *P* *>* 0.05, *n* = 15. Values are expressed as mean ± SD.

All groups of mice except the sham-operated group showed a significant drop in blood flow during surgery, and we analysed the drop in blood flow after monofilament insertion compared to the preoperative baseline blood flow (*P* < 0.01), the percentage decrease in blood flow immediately after middle cerebral artery embolization in each group was as follows: Sham: 0%, ICA1: (86.91 ± 3.47)%, ICA2: (86.48 ± 4.09)%, ECA1: (87.96 ± 3.39)%, ECA2: (86.79 ± 42.5)%, no statistically significant difference between the 4 groups (*P* > 0.05) (Figure 3(a)). It was suggested that the insertion of the monofilament had an embolic effect on the main trunk of the middle cerebral artery in all four groups of mice. During ambulatory blood flow monitoring for 80 min, the percentage decrease in MCA blood flow in each group of mice was as follows: Sham: 0%, ICA1: (47.41 ± 8.28)%, ICA2: (81.17 ± 4.72)%, ECA1: (46.07 ± 3.15)%, ECA2: (81 ± 3.98)%, Inter-group comparisons suggest a statistical difference only between groups 1 and 2 (*P* *<* 0.05) (Figure 3(b)), Extending the monofilament's Silicone-coated length may result in a sustained disruption of MCA blood flow.

### 3.2. Model Success Rate

**Figure 4:**
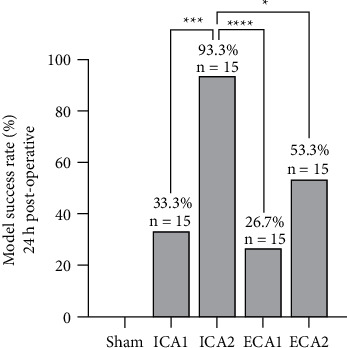
Model success rate for each group, 24 h postoperatively. The success rate of the model in the ICA2 group was higher than in the ICA1, ECA1, and ECA2 groups, and the difference was statistically significant. ^*∗*^*P* *<* 0.05, ^*∗∗∗*^*P* < 0.001, ^*∗∗∗∗*^*P* < 0.0001, *n* = 15.

The success of the model was judged by the presence of distinct white infarct lesions on TTC staining. 24 h postoperative, the model success rates for each group were: Sham: 0, ICA1: 33.3%, ICA2: 93.3%, ECA1: 26.7%, ECA2: 53.3%. ICA2 group, which used the optimised procedure, had a significantly higher model success rate than the ICA1, ECA1, and ECA2 groups (Figure 4). In the ICA2 group, the infarct foci were distributed in the neocortex, striatum, thalamus, and hippocampus, with a better consistency of infarct areas.

### 3.3. Correlation between Cerebral Infarct Volume Percentage Percentage and Neurological Severity Score (NSS)

**Figure 5:**
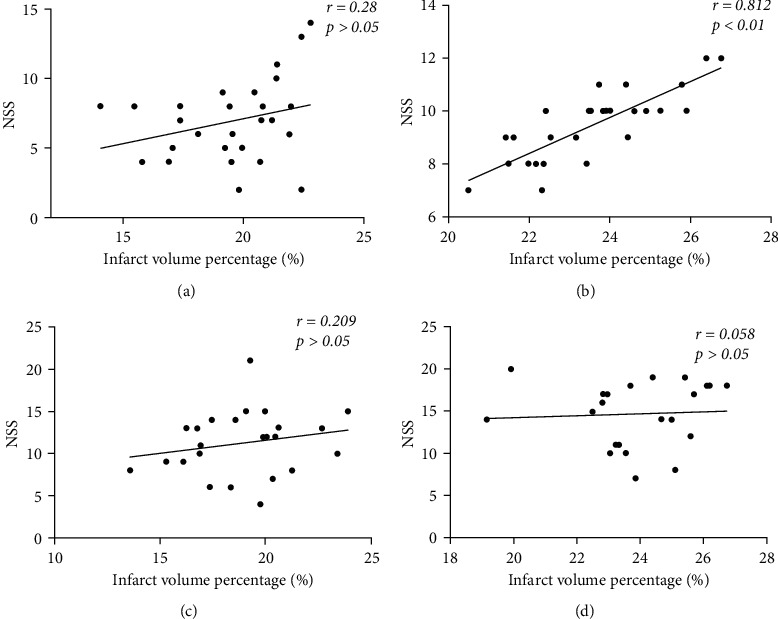
Correlation analysis between NSS and percentage of cerebral infarct volume at 24 h postoperatively. (a) There was no correlation between NSS and the percentage of cerebral infarct volume in the ICA1 group *r* = 0.28, *P* > 0.05, *n* = 15. (b) In the ICA2 group, NSS was significantly correlated with the percentage of cerebral infarct volume *r* = 0.812, *P* < 0.01 *n* = 15. (c) In the ECA1 group, there was no correlation between NSS and percentage of cerebral infarct volume *r* = 0.209, *P* > 0.05, *n* = 15. (d) In the ECA2 group, there was no correlation between NSS and percentage of cerebral infarct volume *r* = 0.0588, *P* > 0.05, *n* = 15.

At 24 h postoperatively, the correlation between NSS and the percentage of infarct volume was analysed. Only the ICA2 group had a strong correlation between NNS and the percentage of cerebral infarct volume, with an optimised surgical approach, the NSS allows for a more accurate assessment of the extent of brain damage (Figure 5).

### 3.4. Postoperative Weight Change

**Figure 6:**
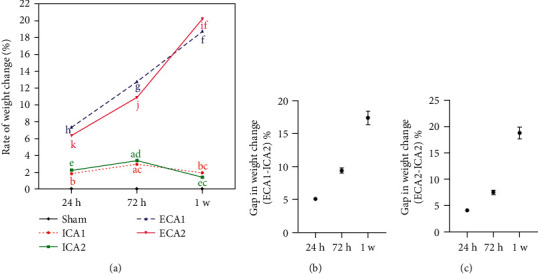
Comparison of rates of weight change, 24 h, 72 h, 1 w postoperatively. (a) Intergroup comparison: at 2 postoperative time points (72 h, 1 w), there was a statistically significant difference in weight change in the ICA2 group compared to ECA1 and ECA2 groups respectively ^*∗∗∗∗*^*P* < 0.0001, ^*∗∗∗∗*^*P* < 0.0001, ^*∗∗∗∗*^*P* < 0.0001, ^*∗∗∗∗*^*P* < 0.0001, *n* = 15. Intragroup comparison: no significant difference in the rate of weight change in the ICA2 group at the 2 postoperative time points (24 h, 1 w) *P* > 0.05, *P* > 0.05, *n* = 15 (b) The difference in weight change between the ECA1 group and the ICA2 group at different time points. (c) The difference in weight change between the ECA2 group and the ICA1 group at different time points. Notes: The letters a, b, c, d, e, f, g, h, i, j, k mark the differences between intergroup and intragroup, the letters are different between the two points suggesting that there is a difference and that the difference is statistically significant, the letters are repeated between the two points suggesting that there is no difference.

The body weights of the mice were measured before, 72 h, 24 h, and 1 w after surgery. At 24 h postoperatively, during intergroup comparison, there was a difference in weight change between the groups, but there is little difference between the groups (Figure 6(a)). At 72 h postoperatively, in intergroup comparison, the change in body weight in the ECA1 and ECA2 groups were significantly greater than that in the ICA2 groups (Figure 6(a)), The difference in weight change between the ICA1 group and ECA1 group and ECA2 group as follows: (9.4 ± 0.4)%, (7.5 ± 0.4)%, ^*∗∗∗∗*^*P* *<* 0.0001, *n* = 15 (Figures 6(b) and 6(c)). At 1w postoperatively, the intragroup comparison suggested that the weight of the ICA2 group recovered to 24 h postoperatively. There was no significant difference in the rate of weight change in the ICA2 group at 24 h and 1 w postoperatively (Figure 6(a)), it was suggested that the body weight of mice in ICA2 groups gradually recovered at 1 week postoperatively. The difference between ICA2 and ECA1 and ECA2 further increased, respectively: (17.4 ± 1.0)%, (18.8 ± 1.1)%, ^*∗∗∗∗*^*P* *<* 0.0001, *n* = 15 (Figures 6(b) and 6(c)). The mice in the ECA1 and ECA2 groups did not recover their feeding ability and gradually lost weight at 1 week postoperatively. With the classical operation, the mice showed increasing weight changes after the operation without recovery, and with the optimised operation, the mice showed decreasing weight changes after the operation with gradual weight recovery.

### 3.5. Survival Rates at Different Time Points Postoperatively

**Figure 7:**
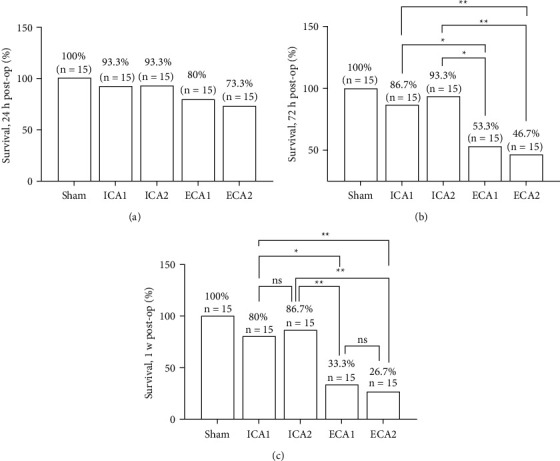
Survival rates between groups at different time points postoperatively. (a) No statistically significant difference in survival rate between groups at 24 h postoperatively *P* > 0.05, *n* = 15. (b) At 72 h postoperatively, the survival rate in the ECA1 group was significantly lower than that in the ICA1 and ICA2 groups, ^*∗*^*P* < 0.05, *n* = 15, the survival rate in the ECA2 group was significantly lower than that in the ICA1 and ICA2 groups ^*∗∗*^*P* < 0.01. *n* = 15 (c) At 1 week postoperatively, there was no difference in survival rates between the ECA1 and ECA2 groups, and similarly between the ICA1 and ICA2 groups, *P* > 0.05, *n* = 15. survival rates in the ICA2 group were significantly higher than those in the ECA1 and ECA2 groups ^*∗∗*^*P* < 0.01. *n* = 15.

The survival rate of the mice was counted at 24 h, 72 h, and 1 w postoperatively. 24 hours postoperatively, with no significant difference in survival between the four groups, but the Survival rates of the ICA1 and ICA2 groups were higher than those of the ECA1 and ECA2 groups at the two postoperative time points (72 h, 1 w), It is worth pointing out that at 24 h and 1 w postoperatively, there was no significant difference in survival rates between the ICA1 and ICA2 groups using the ICA access and between the ECA1 and ECA2 groups using the ECA access (Figure 7). It is suggested that ICA access could reduce the mortality of the mice postoperative.

### 3.6. Wellbeing Assessment

**Figure 8:**
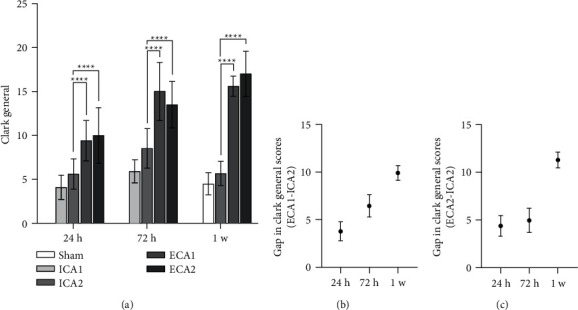
Analysis of Clark's general scores at different postoperative time points. (a) A significant difference in the ICA2 group compared to the ECA1 and ECA2 groups in Clark general scores at 3 postoperative time points 24 h, 72 h, and 1 w. ^*∗∗∗∗*^*P* < 0.0001, *n* = 15, (b) difference in Clark general scores between ICA2 group and ECA1 group at different time points. (c) the difference in Clark general scores between ICA2 and ECA2 groups at different time points.

Clark general scores were performed at 24 h, 72 h, and 1 w postoperatively. The ICA2 group showed a statistically significant difference in the Clark score compared to the ECA1 and ECA2 groups, at three postoperative time points (24 h, 72 h, 1 w) (Figure 8(a)). At 24 h, 72 h, and 1 w postoperatively, the difference between the ICA2 group and the ECA1 and ECA2 groups in Clark general score were 3.8 ± 1.0 and 4.4 ± 1.1, 6.4 ± 1.6 and 5.0 ± 1.3, 10 ± 0.7 and 11.3 ± 0.8 respectively (Figures 8(b) and 8(c)). Subsequently, the gap in Clark general scores increased the longer the postoperative period, whereas there was no gap in the ICA2 group for intergroup comparisons at different times, 1 w after surgery, ICA2 group scores gradually recovered and mice showed an improvement in wellbeing, it is suggested that ICA access can improve well-being in mice by reducing vascular damage.

## 4. Discussion

High mortality and high heterogeneity have been major obstacles in mouse MCAO models [33], resulting in an inability to assess long-term functional outcomes and waste of animals and medicine [34].

Our study focuses on the optimisation of a mouse model of cerebral ischemia-reperfusion. On the one hand, by avoiding damage to the ECA, PPA, and other vessels [24], the survival rate and the wellbeing of the mice are improved by ICA access. Thus, optimising surgical approaches greatly minimizes mortality bias and allows the study of long-term morphological and functional sequelae of stroke in mice. On the other hand, The present study indicated that the consistency and reproducibility of the infarct area of the model was improved by extending the length of the silicone coating, which improved the correlation between behavioural scores and actual brain damage. This method will reduce the number of animals required and provide scientific, economic, and ethical benefits.

The current procedure of ECA access is well established and a simplified method of ligating the CCA has also been proposed. However, ligation of the CCA on one side has a non-negligible effect on haemodynamics, and there is no blood supply to the ECA, PPA, and other vessels on the operated side. Impaired PPA leads to dysphagia and ophthalmoplegia, impaired masticatory muscle function, and feeding difficulties after ECA damage (the results of the present study corroborate this conclusion), both of which increase mortality in mice and resulting discrepancies between behavioural assessments and the actual brain damage [35]. This method is therefore difficult to apply to systematic studies of cerebral ischemia-reperfusion injury. Subsequently, the use of bioadhesive for CCA repair was proposed as a feasible [18], but costly method with negligible damage to the endothelial injury can be followed by massive thrombosis, CCA occlusion, and failure to achieve reperfusion.

During the animal experiments, there are many other factors that affect the success of the mouse brain ischemia-reperfusion model, and the matters related to the surgery are summarized as follows. Select mice with appropriate body weight for surgery; too heavy models have a high failure rate and too light are prone to skull base haemorrhage. It is important to be proficient in the surgical approach. Careful separation of the PPA, ICA, and other vessels is essential for smooth insertion of the monofilament because of the potential for vascular variants at the carotid bifurcation. During the procedure we found abundant reverse blood flow in the PPA, In the classical surgical approach of ECA access, PPA can achieve compensation through the caudal part of the thin monofilament to the middle cerebral artery (MCA) and its surrounding branch arteries, ultimately leading to poor model consistency or even modelling failure [36]. It has been proposed to Permanent suture PPA on top of the classic surgical approach of ECA access, which has branches including the ophthalmic, pterygoid, pharyngeal, and other important branch arteries and is bound to have a serious impact on the well-being of the mice. The optimised surgical approach in this study therefore avoids both compensatory perfusions of the MCA by the PPA and damage to the PPA.

There are various ways to determine the success of a mouse brain ischemia-reperfusion model, noninvasive methods such as neurological deficit scoring, which is too subjective and less accurate, and invasive methods such as TTC staining, which is intuitive but the mice have been executed after the staining is completed, are now widely used for transcranial Doppler flow monitoring. The results of this experiment suggest that by monitoring the cerebral blood flow in the middle cerebral artery of mice, it can be judged that all groups can form effective embolization of the MCA trunk during the operation, but only the ICA2 and ECA2 groups formed a prolonged and effective interruption of blood flow in the middle cerebral artery before the end of the operation, and the ICA2 modelling success rate reached 93.3%. Middle cerebral artery flow is restored in the ICA1 and ECA1 groups, with a modelling success rate of only 26.7%–33.3%, this is still mainly due to the abundant intracranial arterial collateral circulation in mice [37], which cannot be reoperated again even if significant intraoperative MCA blood flow fluctuations are monitored, inevitably resulting in wasted samples. The silicone coating of fine monofilaments is therefore of sufficient length to embolize parts of the ACA and PCoA to reduce collateral compensation is key to increasing the success of moulding and improving the stability of the model.

To further improve the reproducibility and consistency of the mouse ischemia-reinfusion model, the diameter of the silicone monofilaments needs to be further investigated according to the mouse strain, body weight, and intracranial vessel diameter. The number of mice in this experiment is limited and a large sample size will be required to reach a conclusion worthy of recommendation.

## Data Availability

The datasets used and analyzed during the current study are available from the corresponding author upon request.

## References

[1] Caprio F. Z., Sorond F. A. (2019). Cerebrovascular disease. *Medical Clinics of North America*.

[2] Go A. S., Mozaffarian D., Roger V. L. (2014). Heart disease and stroke statistics--2014 update: a report from the American Heart Association. *Circulation*.

[3] Ström J. O., Ingberg E., Theodorsson A., Theodorsson E. (2013). Method parameters’ impact on mortality and variability in rat stroke experiments: a meta-analysis. *BMC Neuroscience*.

[4] Lee S., Hong Y., Park S., Lee S.-R., Chang K.-T., Hong Y. (2014). Comparison of surgical methods of transient middle cerebral artery occlusion between rats and mice. *Journal of Veterinary Medical Science*.

[5] Hata R., Maeda K., Hermann D., Mies G., Hossmann K.-A. (2000). Evolution of brain infarction after transient focal cerebral ischemia in mice. *Journal of Cerebral Blood Flow and Metabolism*.

[6] Yao H., Ibayashi S., Anaga-Akiyoshi F., Fukuda K., Fujishima M. (2001). [YAG laser-induced reperfusion of photothrombotic middle cerebral artery occlusion in rats]. *Nō to shinkei = Brain and nerve*.

[7] Ansar S., Chatzikonstantinou E., Wistuba-Schier A. (2014). Characterization of a new model of thromboembolic stroke in C57 black/6J mice. *Translational Stroke Research*.

[8] Schunke K. J., Toung T. K., Zhang J. (2015). A novel atherothrombotic model of ischemic stroke induced by injection of collagen into the cerebral vasculature. *Journal of Neuroscience Methods*.

[9] Chen Y., Zhu W., Zhang W., Libal N. A novel mouse model of thromboembolic stroke. *J Neurosci Methods*.

[10] Ren M., Lin Z.-J., Qian H. (2012). Embolic middle cerebral artery occlusion model using thrombin and fibrinogen composed clots in rat. *Journal of Neuroscience Methods*.

[11] Fan X., Qiu J., Yu Z. (2012). A rat model of studying tissue-type plasminogen activator thrombolysis in ischemic stroke with diabetes. *Stroke*.

[12] Ringelstein E. B., Biniek R., Weiller C., Ammeling B., Nolte P. N., Thron A. (1992). Type and extent of hemispheric brain infarctions and clinical outcome in early and delayed middle cerebral artery recanalization. *Neurology*.

[13] Ansari S., Azari H., McConnell D. J., Afzal A., Mocco J. (2011). Intraluminal middle cerebral artery occlusion (MCAO) model for ischemic stroke with laser Doppler flowmetry guidance in mice. *Journal of Visualized Experiments*.

[14] Shimamura N., Ohkuma H. (2010). Novel rat middle cerebral artery occlusion model : trans-femoral artery approach combined with preservation of the external carotid artery. *Journal of Neuroscience Methods*.

[15] Cai Q., Xu G., Liu J. (2016). A modification of intraluminal middle cerebral artery occlusion/reperfusion model for ischemic stroke with laser Doppler flowmetry guidance in mice. *Neuropsychiatric Disease and Treatment*.

[16] Morris G. P., Wright A. L., Tan R. P., Gladbach A., Ittner L. M., Vissel B. (2016). A comparative study of variables influencing ischemic injury in the longa and koizumi methods of intraluminal filament middle cerebral artery occlusion in mice. *PLoS One*.

[17] Connolly E. S., Winfree C. J., Stern D. M., Solomon R. A., Pinsky D. J. (1996). Procedural and strain-related variables significantly affect outcome in a murine model of focal cerebral ischemia. *Neurosurgery*.

[18] Trotman-Lucas M., Kelly M. E., Janus J., Fern R., Gibson C. L. (2017). An alternative surgical approach reduces variability following filament induction of experimental stroke in mice. *Disease Models and Mechanisms*.

[19] Koizumi J.-i., Yoshida Y., Nakazawa T., Ooneda G. (1986). Experimental studies of ischemic brain edema. *Nosotchu*.

[20] Longa E. Z., Weinstein P. R., Carlson S., Cummins R. (1989). Reversible middle cerebral artery occlusion without craniectomy in rats. *Stroke*.

[21] Mccoll B. W., Carswell H. V., Mcculloch J., Horsburgh K. (2004). Extension of cerebral hypoperfusion and ischaemic pathology beyond MCA territory after intraluminal filament occlusion in C57Bl/6J mice. *Brain Research*.

[22] Smith H. K., Russell J. M., Granger D. N., Gavins F. N. E. (2015). Critical differences between two classical surgical approaches for middle cerebral artery occlusion-induced stroke in mice. *Journal of Neuroscience Methods*.

[23] Dittmar M., Spruss T., Schuierer G., Horn M. (2003). External carotid artery territory ischemia impairs outcome in the endovascular filament model of middle cerebral artery occlusion in rats. *Stroke*.

[24] Dittmar M. S., Vatankhah B., Fehm N. P. (2005). The role of ECA transection in the development of masticatory lesions in the MCAO filament model. *Experimental Neurology*.

[25] Trueman R. C., Harrison D. J., Dwyer D. M., Dunnett S. B., Hoehn M., Farr T. D. (2011). A critical Re-examination of the intraluminal filament MCAO model: impact of external carotid artery transection. *Translational Stroke Research*.

[26] Lourbopoulos A., Mamrak U., Roth S. (2017). Inadequate food and water intake determine mortality following stroke in mice. *Journal of Cerebral Blood Flow and Metabolism*.

[27] Kaawa K. (1998). Cerebral ischemia after bilateral carotid artery occlusion and intraluminal suture occlusion in mice: evaluation of the patency of the posterior communicating artery. *Journal of Cerebral Blood Flow and Metabolism*.

[28] Akamatsu Y., Shimizu H., Saito A., Fujimura M., Tominaga T. (2012). Consistent focal cerebral ischemia without posterior cerebral artery occlusion and its real-time monitoring in an intraluminal suture model in mice. *Journal of Neurosurgery*.

[29] Ansari S., Azari H., Mcconnell D. J., Afzal A., Mocco J. (2011). Intraluminal middle cerebral artery occlusion (MCAO) model for ischemic stroke with laser Doppler flowmetry guidance in mice. *Journal of Visualized Experiments*.

[30] Chen J., Sanberg P. R., Li Y. (2001). Intravenous administration of human umbilical cord blood reduces behavioral deficits after stroke in rats. *Stroke*.

[31] Swanson R. A., Sharp F. R. (1994). Infarct measurement methodology. *Journal of Cerebral Blood Flow and Metabolism*.

[32] Clark W. M. (1997). Monofilament intraluminal middle cerebral artery occlusion in the mouse. *Neurological Research*.

[33] Aspey B. S., Taylor F. L., Terruli M., Harrison M. J. G. (2000). Temporary middle cerebral artery occlusion in the rat: consistent protocol for a model of stroke and reperfusion. *Neuropathology and Applied Neurobiology*.

[34] Rosell A., Agin V., Rahman M. (2013). Distal occlusion of the middle cerebral artery in mice: are we ready to assess long-term functional outcome. *Transl Stroke Res*.

[35] Bieber M., Gronewold J., Scharf A.-C. (2019). Validity and reliability of neurological scores in mice exposed to middle cerebral artery occlusion. *Stroke*.

[36] Chen Y., Ito A., Takai K., Saito N. (2008). Blocking pterygopalatine arterial blood flow decreases infarct volume variability in a mouse model of intraluminal suture middle cerebral artery occlusion. *Journal of Neuroscience Methods*.

[37] Hua Z., Prabhakar P., Sealock R. (2010). Faber JEWide genetic variation in the native pial collateral circulation is a major determinant of variation in severity of stroke. J Cereb Blood Flow Metab 30:923-934. *Journal of the International Society of Cerebral Blood Flow & Metabolism*.

